# Supplementary material for: Leaf Age-Dependent Photosystem II Photochemistry and Oxidative Stress Responses to Drought Stress in Arabidopsis thaliana Are Modulated by Flavonoid Accumulation
Source: Molecules. 2021 Jul 8;26(14):4157. doi: 10.3390/molecules26144157 (PMC8307756; doi:10.3390/molecules26144157)
Supplement: Supplementary file 1 [file molecules-26-04157-s001.zip › molecules-1273366-SI.pdf]

molecules

Supplementary Information

Leaf Age-Dependent Photosystem II Photochemistry and Oxidative Stress Responses to Drought Stress in *Arabidopsis thaliana* are Modulated by Flavonoid Accumulation

Ilektra Sperdouli[1,2], Julietta Moustaka[1,3], Georgia Ouzounidou[4] and Michael Moustakas[1*]

[1] Department of Botany, Aristotle University of Thessaloniki, GR-54124 Thessaloniki, Greece; moustak@bio.auth.gr
[2] Present address: Institute of Plant Breeding and Genetic Resources, Hellenic Agricultural Organization-Demeter (ELGO-Dimitra), Thermi, 57001 Thessaloniki, Greece; ilektras@bio.auth.gr
[3] Present address: Department of Plant and Environmental Sciences, University of Copenhagen, Thorvaldsensvej 40, DK-1871 Frederiksberg C, Denmark; moustaka@plen.ku.dk
[4] Institute of Food Technology, Hellenic Agricultural Organization-Demeter (ELGO-Dimitra), 1 S. Venizelou Str., GR-14123 Lycovrissi, Greece; geouz@nagref.gr
* Correspondence: moustak@bio.auth.gr

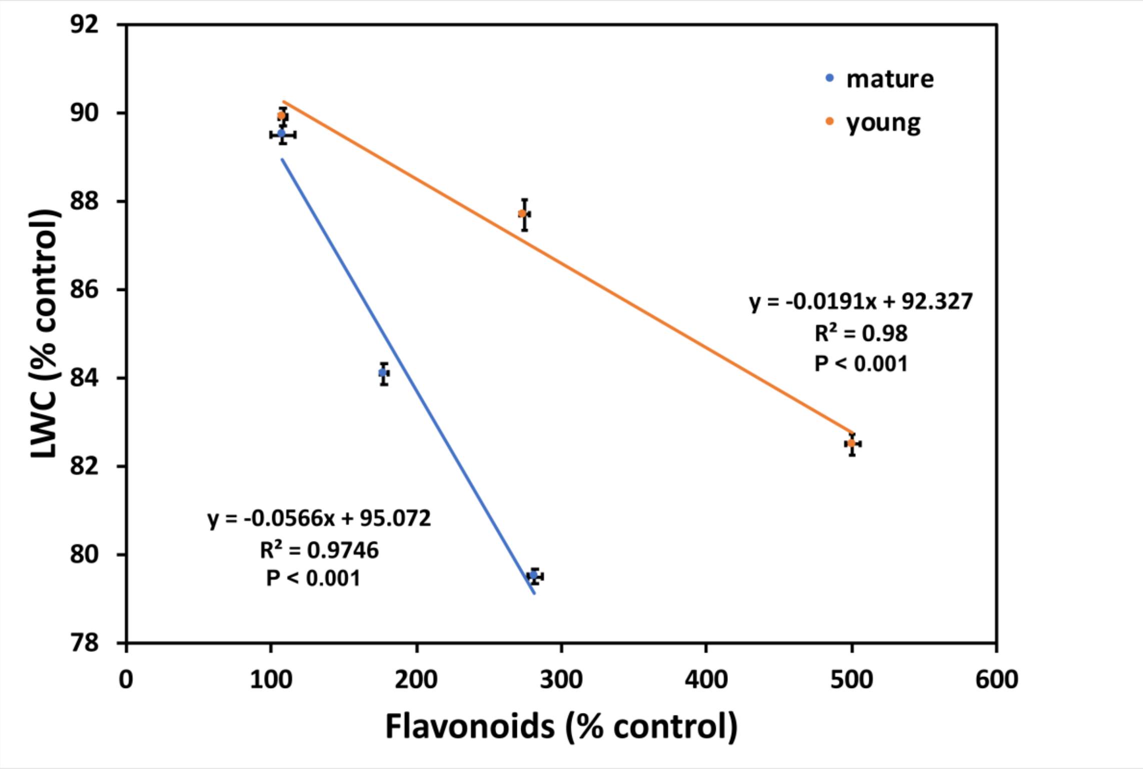

Figure S1. The relationship between leaf water content and flavonoid accumulation expressed as percentage of control (well-watered plants), in both *A. thaliana* young leaves and mature leaves at the three DS treatments. Error bars represent ± standard error of the mean (n = 5–6).
